# Trends in Computed Tomography Utilization and Association with Hospital Outcomes in a Chinese Emergency Department

**DOI:** 10.1371/journal.pone.0040403

**Published:** 2012-07-12

**Authors:** Jian-Cang Zhou, San-Wei Zheng, Yun-Xian Yu, Keith Rouleau, Wei-Liang Jiang, Chong-Wu Jin, Dao-Yang Zhou, Kong-Han Pan, Yun-Song Yu

**Affiliations:** 1 Department of Critical Care Medicine, Sir Run Run Shaw Hospital, Zhejiang University School of Medicine, Hangzhou, Zhejiang, China; 2 Department of Biomedical Informatics, Sir Run Run Shaw Hospital, Zhejiang University School of Medicine, Hangzhou, Zhejiang, China; 3 Department of Epidemiology and Health Statistics, College of Public Health, Zhejiang University, Hangzhou, Zhejiang, China; 4 Vallejo, California, United States of America; 5 Department of Radiology, Sir Run Run Shaw Hospital, Zhejiang University School of Medicine, Hangzhou, Zhejiang, China; 6 Department of Infectious Disease, Sir Run Run Shaw Hospital, Zhejiang University School of Medicine, Hangzhou, Zhejiang, China; Yale University School of Medicine, United States of America

## Abstract

**Background:**

Excessive use of computed tomography (CT) in emergency departments (EDs) has become a concern due to its expense and the potential risks associated with radiation exposure. Although studies have shown a steady increase in the number of CT scans requested by ED physicians in developed countries like the United States and Australia, few empirical data are available regarding China.

**Methods and Findings:**

We retrospectively analyzed a database of ED visits to a tertiary Chinese hospital to examine trends in CT utilization and their association with ED outcomes between 2005 and 2008. A total of 197,512 ED visits were included in this study. CT utilization increased from 9.8% in 2005 to 13.9% in 2008 (*P*<.001 for trend). The ED length of stay for visits with CT utilization was 0.6 hour longer than those in which CT was not obtained. CT utilization increased the ED cost by an average $48.2. After adjustment for patients’ demographics, arrival hours and clinical condition, CT utilization during ED visits was significantly associated with high ED cost (Odds Ratio [OR]: 21.70; 95% confidence interval [CI], 17.00–27.71), long ED length of stay (OR: 1.22; 95%CI, 1.12–1.34), and more likely to receive emergency operations (OR: 2.31; 95%CI, 1.94–2.76). However, there was no significant correlation between CT use and the possibility to be admitted to inpatient wards (OR: 0.82; 95%CI, 0.65–1.04). With respect to the time-related trends, CT utilization during ED visits in all study years was significantly associated with high ED cost and more likely to receive emergency operations.

**Conclusion:**

CT utilization was associated with higher ED cost, longer ED length of stay and more likely to receive emergency operations, but did not correlate with a significant change in the admission rate.

## Introduction

Emergency department (ED) physicians are often challenged to correctly manage and triage patients in a timely fashion, deciding whether the patient needs emergency surgery, requires hospital admission for further workup, or can be safely discharged from the hospital [1]. The widespread accessibility and associated diagnostic superiority of computed tomography (CT) have made it an integral diagnostic tool in the evaluation of patients presenting to EDs [2].

Studies in the United States (US) and Australia [2]–[5] have shown a steady increase in the number of CT scans requested by ED physicians although use of CT is associated with increased cancer risk due to radiation exposure [6], [7]. The increasing use of CT has become a subject of concern for patients, health care providers and regulators, and is receiving increased attention in the medical literature and popular media [2]. A difficult balance must be achieved between making a timely diagnosis and minimizing the use of medical radiation. Given the fact that CT scans are expensive and national health care budgets are limited in developing countries, we postulated that the rate of increase of CT utilization in these countries maybe slower than that in developed countries, yet few empirical data are available regarding this.

In this study, we primarily sought to determine the trends in CT utilization during ED visits in a Chinese tertiary teaching hospital over a four-year period. Secondly, we planned to explore whether CT use was associated with the ED outcomes.

## Materials and Methods

### Study Design and Setting

The study was a retrospective review of ED visits to Sir Run Run Shaw Hospital (SRRSH), an urban, 800-bed major tertiary teaching hospital in Hangzhou, China, from January 1, 2005 to December 31, 2008, to examine the trends in CT utilization and their association with ED outcomes. The institutional review board of SRRSH approved the study protocol and waived from the need for a consent form.

Since its opening in 1994, SRRSH has been closely cooperating with the Loma Linda University in California. The establishment and development of the emergency department involved significant assistance from US physicians [8]. In 2006, it became the first public hospital in mainland China to be accredited by the Joint Commission International, a US-based, World Health Organization-authorized organization for medical quality evaluation. All emergency physicians are required to attend formal training programs in the intensive care unit (ICU) every 6 months, where they improve patient evaluation skills and practice protocols for scenarios like sepsis [9]. Our emergency department is divided into three separate areas: minor trauma, internal medicine and resuscitation area. These are usually staffed with residents, fellow physicians, and senior fellows or attending physicians respectively. All the triage category 1 and vast majority of triage category 2 on Emergency Severity Index, which is a 5-level triage system with category 1 represents the sickest or most urgent cases [10], were triaged to the resuscitation area. Because of perceived inadequacies of Chinese primary medical care, patients frequently prefer to attend tertiary teaching hospitals rather than family or community services even for minor problems like upper respiratory infection or gastrointestinal dysfunction. Therefore, although many Chinese EDs are very busy, the average admission rate is markedly lower than EDs in developed countries [9].

During the study period, non-enhanced CT examination was available 24 hours a day, seven days a week. However, urgent contrast-enhanced CT could only be organized after a radiologist validated the CT request. A plain CT scan cost around $34 per body part, while a contrast-enhanced CT cost ranged from $88 to $105 according to the type of contrast used. Before the CT examinations, informed consent was obtained and every women of childbearing age was checked whether she was pregnant. CT images were simultaneously transferred to radiologists and emergency physicians through the picture archiving and communication systems (PACS), and a formal written report was prepared within thirty minutes after CT scan. During the study period, the CT hardware at our institution unchanged. The CT examinations were performed with either a single–detector row scanner (HighSpeed CT/i; GE Healthcare), or a 16-detector row scanner (SOMATOM, Sensation 16; Siemens). The CT examination protocols were established by the radiologists, and the specific scanning parameters varied depending on the scanner used.

### Data Sources and Processing

The hospital’s information system was queried to extract all the ED visits during the study period. We excluded those visits staffs used ED to prescribe medication for themselves, or the ED was utilized as a buffer of hospital overflow to board some elective admissions. For each visit, the following data elements were extracted: (1) date and time of registration; (2) demographic characteristics (age, gender); (3) triage location; (4) ID of the ED provider; (5) whether the patient underwent a CT scan, and the type of CT use; (6) disposition location; (7) whether the patient underwent an emergency operation; (8) date and time of transfer to inpatient ward; (9) ED cost. In SRRSH, 8∶00 AM and 5∶00 PM during weekdays was defined as office hours. Although triage categories were stuck to the charts for every ED visits, this information was not electronic data and can not be extracted retrospectively from the hospital’s information system. Therefore, triaged to the resuscitation was used as a surrogate for the clinical condition.

### Definition of CT Utilization and ED Outcomes

ED visits met the abovementioned inclusion criteria and in which patients underwent any kind of CT scans were defined as CT utilization during ED visits. CT scans were classified by body part into five groups: “head,” “cervical,” “chest,” “abdomen”, and “miscellaneous.” Patients underwent several CT scans on one occasion were recorded separately. ED length of stay (ED LOS) was calculated as the difference between the time of registration and time of the patient departure from the ED. Since the exact departure time of discharged ED visits was not easily to record, we only calculated the ED LOS for admitted patients. ED cost was calculated as all the fees charged during patients’ stay in ED. Emergency admission was defined as the ED visits were admitted either to inpatient wards or to intensive care units. An emergency operation was defined as surgical procedures that patients were transferred from ED to the operating theatre directly.

### Primary Data Analysis

Descriptive data were reported as either mean ± SD, median (interquartile range) or number and percentage. Multiple comparison analysis between study years was performed using log-linear analysis for categorical data and analysis of variance for normally distributed continuous data. With respect to the differences in ED outcomes between visits with or without CT utilization, categorical variables were compared using chi-square analysis. Continuous variables were compared using Independent Sample *T* test for normally distributed data and Mann-Whitney *U* test for non-normally distributed data. Since SRRSH only treats for patients older than fourteen, age was classified into the following groups: 14 to 29, 30 to 49, 50 to 69, and ≥70 years. The working years of the CT ordering provider was used as a surrogate to assess the effect of clinical experience on the frequency of CT use. Usually, in SRRSH, a resident works three years to be a fellow and eight years to be an attending. Therefore, we categorized the working years of the physicians into ≤3, 3–8 and ≥8 years categories. Since one of the main focus of the analysis was to examine whether CT utilization associated with better or worse outcomes for ED visits, the median values of the ED cost and ED LOS were used as cutoffs to transform the data into categorical variables (high/low ED cost, and long/short ED LOS respectively) for regression analysis.

To identify potential correlation between CT utilization and ED outcomes, binary logistic regression analysis was performed using ED outcomes (high ED cost (yes/no), long ED LOS (yes/no), admitted to wards (yes/no), and received emergency operations (yes/no) respectively) as the dependent variable and CT utilization, patient demographics, arrival hours and clinical condition as the independent variables. The generalized estimating equation (GEE) regression model was used to account for the effect of clustering of patients among physicians, which may make their observations not independently. Odds ratios and their 95% confidence intervals (95% CIs) were calculated. Statistical analysis was performed, using SAS version 9.1 (SAS Institute, Cary, NC) and SPSS 16.0 (Chicago, Ill, USA). Significance was defined as a *P* value <.05.

## Results

### Sample Characteristics

Although there were a total of 214,323 ED visits during the study period, the study cohort comprised 197,512 visits, 23,899 of them received a total 24,954 CTs. The study period witnessed an approximate 30% increase in the number of ED visits, from 43,982 to 56,516. Mean age was 38.3 years, 52.9% were male; 44.7% visited within office hours. Patients aged 14–29 accounted for 37.3%, 30–49 group 40.0%, 50–69 group 16.4%, and those ≥70 6.3%. With respect to experience of ED providers, physicians working ≤3 years saw 42.4% of total ED visits, physicians working 3–8 years saw 46.8%, and physicians working ≥8 years saw 10.8%.

### Trends in CT Utilization

**Figure 1:**
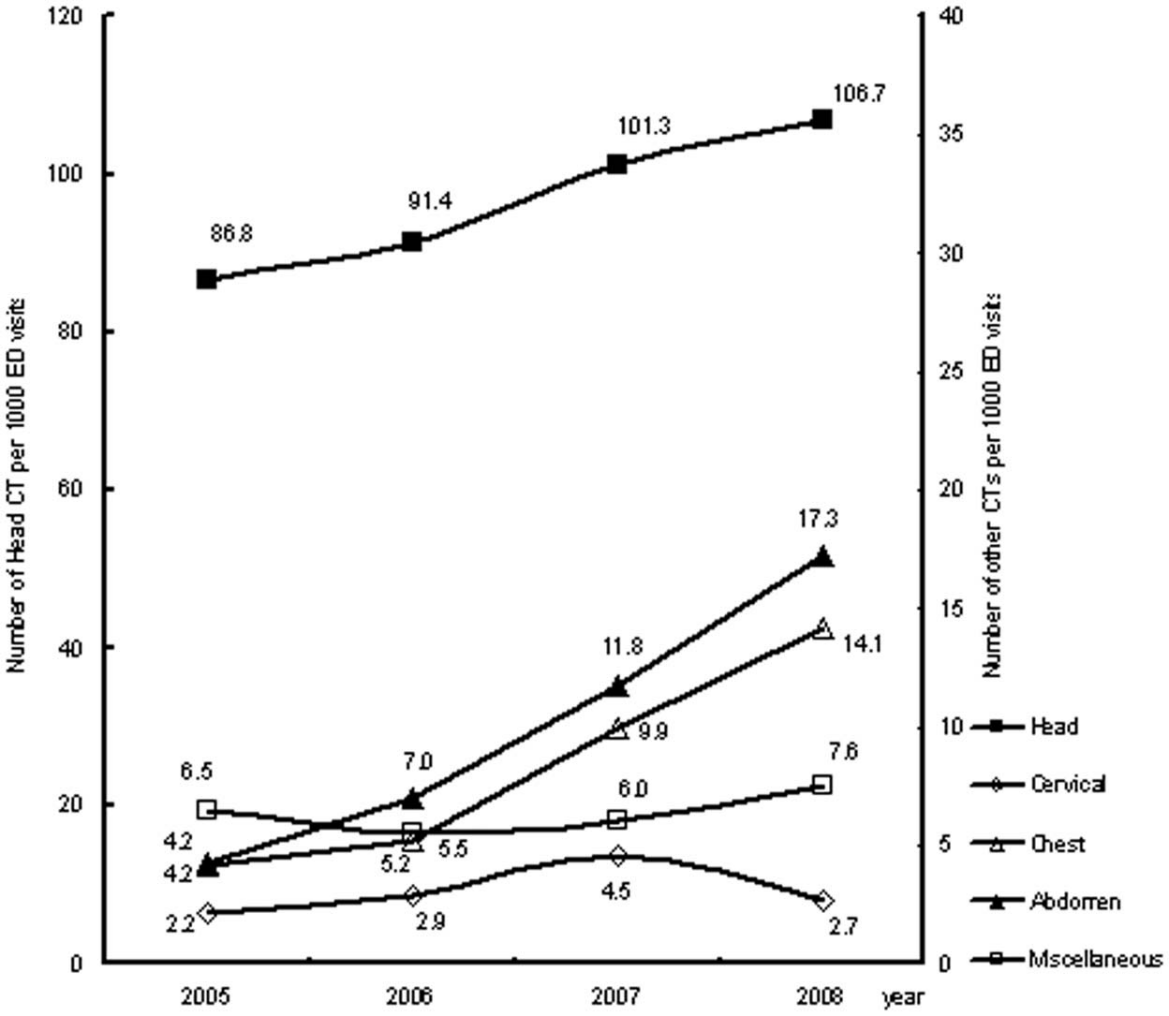
Trends in CT utilization per 1000 emergency department visits by CT type from 2005–2008.

There was a significant increase of CT utilization rate during the study period from 9.8% to nearly 14% (*P*<.001 for the trend, Table 1). The demographics of the study patients demonstrated significant difference within the study years. The percentages of patients arrived at office hours and triaged to the resuscitation area remained similar although there were significant differences throughout the four study years (Table 1). Numerical data of CT per 1000 ED visits by CT type and year was shown in Figure 1. During this period, the rate of visits underwent head CT increased by 23%, cervical CT by 26%, chest CT by 238%, abdominal CT by 310%, and miscellaneous CT by 16%.

**Table 1:** Patient characteristics for emergency department visits from 2005 to 2008.

Variables	2005	2006	2007	2008	Overall	*P*
	(n = 43,982)	(n = 47,970)	(n = 49,044)	(n = 56,516)	(n = 197,512)	
ED visits with CT utilization (%)	4332 (9.8%)	5294 (11.0%)	6418 (13.1%)	7855 (13.9%)	23,899 (12.1%)	<.0001
Male gender (%)	24259(55.2%)	25788(53.8%)	25577(52.2%)	28850(51.0%)	104,474 (52.9%)	<.0001
Age, yrs	37.7±16.0	37.3±16.1	37.8±16.3	39.9±17.3	38.3±16.5	<.0001
Office hours arrival (%)	19,546(44.4%)	21,088(44.0%)	21,766(44.4%)	25,959(45.9%)	88,359 (44.7%)	<.0001
Triaged to the resuscitation area (%)	3519(8.0%)	3656(7.6%)	3707(7.6%)	4178(7.4%)	15,060 (7.6%)	.004

ED, emergency department, CT, computed tomography.

### Association between CT Utilization and ED Outcomes

Of the entire sample, 9.4% or 18,608 were admitted to the hospital, including 4488, or 2.3%, who were admitted to ICU. Among the entire sample, CT use was higher in progressively older age categories. ED visits with CT utilization were more likely to be male, more critically ill, and to arrive during office hours. As expected, physicians with less experience, less than 3 years, ordered CTs on a fairly large number of their patients, at 13.6%. That number dropped by more than half, to 6.6%, in physicians who had between three and eight years of experience. However, there was a dramatic increase in the rate of CT use (30.1%) for physicians with more than eight years of experience, more than doubling that of the inexperienced physicians (Table 2).

**Table 2:** CT utilization rate for emergency department visits.

Covariates	Categories	ED Visits (in thousands)	Received CT
Gender	Male	104.5	14.3%
	Female	93.0	9.7%
Age, yrs	14–29	73.6	8.5%
	30–49	79.0	12.3%
	50–69	32.3	16.3%
	≥70	12.5	21.1%
Office hours arrival	Yes	88.4	13.4%
	No	109.2	11.1%
Physicians’ working years	≤3	83.7	13.6%
	3–8	92.4	6.6%
	≥8	21.3	30.1%
Triaged to the resuscitation area	Yes	15.1	37.3%
	No	182.5	10.0%

ED, emergency department, CT, computed tomography.

In the univariate analysis, the ED LOS for visits with CT utilization was 0.6 hour longer than those in which CT was not obtained. Moreover, CT utilization increased the ED cost by an average $48.2, and was associated more likely to be admitted or to receive emergency operations (Table 3). The median ED cost for each study year was $28.9, $21.1, $21.5, and $25.4 respectively. While the median ED LOS for every study year was 10.7, 11.1, 11.0 and 10.5 hours respectively. Thus, the ED cost and ED LOS were dichotomized into high (≥ median) and low ED cost, and long (≥ median) and short ED LOS respectively. The association between CT utilization and ED outcomes were further assessed by regression analysis with adjustment for age, patient gender, arrival hours and clinical condition (Table 4). CT utilization during ED visits was significantly associated with high ED cost (OR: 21.70; 95%CI, 17.00–27.71), long ED LOS (OR: 1.22; 95%CI, 1.12–1.34), and more likely to receive emergency operations (OR: 2.31; 95%CI, 1.94–2.76). However, there was no significant correlation between CT use and the possibility to be admitted to inpatient wards (OR: 0.82; 95%CI, 0.65–1.04). To determine the association between CT utilization and ED outcomes year by year over the study period, binary logistic regression analysis was performed using annual ED outcomes as the dependent variable and CT utilization, patient demographics, arrival hours and clinical condition as the independent variables (Table 5). CT utilization during ED visits in all study years was significantly associated with high ED cost and more likely to receive emergency operations. Although CT use during ED visits in 2006 and 2008 was associated with long ED LOS, CT utilization was not significantly correlated with ED LOS in 2005 and 2007. While CT use in 2007 was associated with less likely to be admitted, there were no significant association between CT utilization and whether they were admitted to inpatient wards in other three years (Table 5).

**Table 3:** Outcomes for emergency department visits with/without CT utilization.

Variables	ED visits with CT utilization	ED visits without CT utilization	*P*
	(n = 23,899)	(n = 173,613)	
ED cost ($)	67.5 (39.5∼116.3)	19.3 (7.5∼42.5)	<.0001
ED length of stay (hr)	11.3 (8.2∼15.8)	10.7 (7.8∼15.5)	.0003
Admitted to wards (%)	4167 (17.4%)	14,441 (8.3%)	<.0001
Received emergency operations (%)	1196 (5.0%)	1748 (1.0%)	<.0001

ED, emergency department, CT, computed tomography.

**Table 4:** Association between CT utilization and ED outcomes†.

ED outcomes	CT utilization	OR		95% CI	*P*	
High ED cost	No		Reference	–	–	
	Yes (24.0%)		21.70	17.00–27.71	<.0001	
Long ED length of stay	No		Reference	–	–	
	Yes (24.1%)		1.22	1.12–1.34	<.0001	
Admitted to wards	No		Reference	–	–	
	Yes (22.4%)		0.82	0.65–1.04	.1045	
Received emergency operations	No		Reference	–	–	
	Yes (40.6%)		2.31	1.94–2.76	<.0001	

† Regression model adjusted for age, patient gender, arrival hours, and clinical condition. General estimation equation (GEE) was used to account for the clustering of patients among physicians.

ED, emergency department, OR, Odds Ratio, CI, confidence interval.

**Table 5:** Association between CT utilization and ED outcomes for ED visits by study year†.

ED outcomes	Studyyear	OR	95% CI	*P*
High ED cost	2005	13.03	9.66–17.57	<.0001
	2006	56.41	40.85–77.89	<.0001
	2007	57.14	46.58–70.08	<.0001
	2008	13.35	10.06–17.71	<.0001
Long ED length of stay	2005	1.12	0.98–1.28	.1047
	2006	1.42	1.20–1.69	<.0001
	2007	1.06	0.92–1.22	.4034
	2008	1.32	1.10–1.59	.0035
Admitted to wards	2005	0.82	0.59–1.15	.2488
	2006	0.63	0.38–1.04	.0737
	2007	0.64	0.43–0.94	.0248
	2008	0.99	0.66–1.47	.9444
Received emergency operations	2005	2.63	2.14–3.23	<.0001
	2006	2.42	1.77–3.31	<.0001
	2007	2.32	2.16–2.50	<.0001
	2008	2.06	1.50–2.83	<.0001

† Regression model adjusted for age, patient gender, arrival hours, and clinical condition. General estimation equation (GEE) was used to account for the clustering of patients among physicians.

ED, emergency department, OR, Odds Ratio, CI, confidence interval.

## Discussion

This study demonstrated remarkable increase of CT use during ED visits over 4 years in a Chinese tertiary hospital. To the best of our knowledge, this is the first study to document a trend of CT use in China. In 2008, 13.9% of ED visits received CT scans, compared to 9.8% in 2005. Although the increasing CT utilization did not correlate with a significant change of admission rate of ED visits, it was associated with higher ED cost, longer ED LOS and higher possibility to receive emergency operations.

Surprisingly, the trend of CT use in SRRSH was comparable to that in developed countries [2], [3]. Moreover, we did not find evidence that the increased use of CT in the ED was tapering by 2008. Our finding that CT was used more frequently in older patients than in younger patients is similar to the findings of other authors [3]. Factors that promote the increasing use of CT have been enumerated by others [3], [11]. The ED is a high-risk environment where often test results of patients without classic signs and symptoms are sometimes unexpectedly positive [11]. In China, due to the trend towards worsening physician-patient relationships [12], there are significant trust issues. These have lead to increased demands by the patient for doctors to perform tests and prescribe medications. Also, they may have lead to and increased tendency for the physicians to give in to the patient. From the ED providers’ perspective, the cost-effectiveness analysis of ordering CT scans for patients so as to avoid malpractice as much as possible seems simple, even in low probability scenarios. This is confirmed by our study that junior ED providers, with less than three years experience, although they tend to have lower-acuity patients and less significant trauma patients, had a higher CT utilization compared to senior ones. The finding that there was a dramatic increase of CT use for ED providers with more than eight years may reflect the fact their patients may be more severely ill or injured, requiring additional, even whole-body CT scans so as to help evaluate patient situation as soon as possible. Other additional factors that may also increase CT utilization include physicians’ lack of knowledge of radiation risk, superior diagnostic advantages of CT, and other physicians outside EDs using EDs as an urgent testing center [11].

The increasing rates of CT use have called into question the medical appropriateness of these examinations. According to the US Government Accountability Office, annual spending on CT imaging swelled from $975 million in 2000 to $2171 million in 2007. Our study demonstrated that CT utilization increased the ED cost by an average $48.2. Therefore, whatever combination of factors is responsible for the increase in CT utilization for ED visits, the increase should ultimately be justified by improving health outcomes. Injury-related ED visits during which CT or MRI was obtained lasted 126 minutes longer than those without CT or MRI [4]. In our study, on average, visits with CT utilization stayed 36 minutes longer in ED than for visits in which CT was not obtained. After adjustment for covariates, CT utilization was significantly associated with high ED cost (OR: 21.70) and long ED length of stay (OR: 1.22). A prior study demonstrated increase in CT use for patients with abdominal pain without any increase in the rate of diagnosis of significant intra-abdominal conditions [11]. For ED visits with suspected urinary tract calculi, in spite of CT use increase, there was no change in health outcomes as measured by rates of initial or subsequent hospital admission, return visits to the ED, or frequency of subsequent hospitalization for abdominal symptoms [13]. Although CT utilization did not correlate with change of admission rate in the study, CT utilization during ED visits was associated with more likely to have emergency operations. In our study, head CT accounted for the vast majority of the total CT utilization. However, while some authors have advocated performing whole-body CT scanning in the setting of trauma [14], [15], other authors have addressed the medical appropriateness of head CT for minor head injury through the publication of imaging guidelines [16]–[18].

Given the fact appropriate utilization of CT could yield potential benefit in both economic savings and reduced radiation exposure, we propose the following recommendations. First and foremost, since ED physicians and patients had been found to underestimate the CT radiation risk [19], [20], media like posters, and television could be used in the ED, radiology department to make current information regarding the magnitude of CT radiation dose and possible long-term consequences more available, both to physicians and patients. Secondly, since many physicians cited the avoidance of malpractice as the culprit for the excessive CT testing even in a low probability scenario, urgent medical reform and administration support are necessary to improve the physician-patient relationship, and to keep physicians from defensively ordering CT scans for any minor diseases [11]. Furthermore, alternative diagnostic strategies should be considered whenever possible, including ultrasonography, and MRI. If the alternative investigation is clearly diagnostic, then the patient is spared substantial radiation. Hence, CT should be reserved for circumstances in which diagnosis is still equivocal and there is reasonable index of suspicion. More importantly, both the clinician and the radiologist must shoulder the responsibility to assess whether the benefit to the patients is substantially greater than the risk associated with the radiation. Finally, since repeated CT scans are common during transfer or visits to different EDs, a centralized system of medical imaging storage needs to be implemented, to make imaging data more readily available to the treating physicians, while still protecting patient confidentiality [5].

Our study has several limitations. First, we demonstrated the findings by retrospective data review in a single institution and a particular country, which may raise concerns about the generalizability of the results. Healthcare systems of various countries differ greatly. However, as reported by other authors [4], [11], [13], we demonstrated that the significantly increased CT utilization rate during ED visits was associated with an increased ED cost and length of stay, but did not lead to a significant change of admission rate. Second, additional information related to ED visits maybe informative because external causes of increased CT use, such as increasing acuity of patients and/or direct clinical referrals to the ED specifically for CT may influence the rate of CT use. Given that the study was a retrospectively analysis, we can not provide all these information. Third, some very junior physicians may occasionally use the access ID of senior colleagues to give orders because some medication may be only accessible to senior physicians in our hospital, this may influence the coding of physicians’ ID in GEE regression.

In summary, CT utilization was associated with higher ED cost, longer ED length of stay and more likely to receive emergency operations, but did not correlate with a significant change of admission rate.
